# *Ex vivo* metabolite profiling of paediatric central nervous system tumours reveals prognostic markers

**DOI:** 10.1038/s41598-019-45900-x

**Published:** 2019-07-19

**Authors:** Christopher D. Bennett, Simrandip K. Gill, Sarah E. Kohe, Martin P. Wilson, Nigel P. Davies, Theodoros N. Arvanitis, Daniel A. Tennant, Andrew C. Peet

**Affiliations:** 1 Institute of Cancer and Genomic Sciences, University of Birmingham, Birmingham, United Kingdom; 2 Birmingham Women’s and Children’s Hospital NHS Foundation Trust, Birmingham, United Kingdom; 3 Birmingham University Imaging Centre, School of Psychology, University of Birmingham, Birmingham, United Kingdom; 4 University Hospitals Birmingham NHS Foundation Trust, Birmingham, United Kingdom; 5 Institute of Digital Healthcare, WMG, University of Warwick, Coventry, United Kingdom; 6 Institute of Metabolism and Systems Research, University of Birmingham, Birmingham, UK

**Keywords:** Paediatric cancer, Metabolomics, CNS cancer, Prognostic markers

## Abstract

Brain tumours are the most common cause of cancer death in children. Molecular studies have greatly improved our understanding of these tumours but tumour metabolism is underexplored. Metabolites measured *in vivo* have been reported as prognostic biomarkers of these tumours but analysis of surgically resected tumour tissue allows a more extensive set of metabolites to be measured aiding biomarker discovery and providing validation of *in vivo* findings. In this study, metabolites were quantified across a range of paediatric brain tumours using ^1^H-High-Resolution Magic Angle Spinning nuclear magnetic resonance spectroscopy (HR-MAS) and their prognostic potential investigated. HR-MAS was performed on pre-treatment frozen tumour tissue from a single centre. Univariate and multivariate Cox regression was used to examine the ability of metabolites to predict survival. The models were cross validated using C-indices and further validated by splitting the cohort into two. Higher concentrations of glutamine were predictive of a longer overall survival, whilst higher concentrations of lipids were predictive of a shorter overall survival. These metabolites were predictive independent of diagnosis, as demonstrated in multivariate Cox regression models. Whilst accurate quantification of metabolites such as glutamine *in vivo* is challenging, metabolites show promise as prognostic markers due to development of optimised detection methods and increasing use of 3 T clinical scanners.

## Introduction

Cancer is a major cause of death from disease in childhood, and brain tumours are the most common cause of cancer-related death in this age group^1^. Whilst some brain tumours now have a very good prognosis, others have continued to present a challenge, highlighting the need for new techniques for investigation and management. Identifying novel biomarkers of prognosis would allow more accurate treatment stratification to improve survival rates and reduce long-term morbidity. A key strategy in optimizing the clinical management of children with brain tumours is to identify subgroups that have prognostic significance, and this has been particularly successful in medulloblastoma where molecular subgroups have already been incorporated into the accepted diagnostic classification^2^. Whilst most molecular markers have been defined by tumour genetics, there is an increasing interest in tumour metabolism as both a biomarker of prognosis and potential therapeutic target. Mutations in the metabolic enzyme isocitrate dehydrogenase (IDH) act as a marker of good prognosis in gliomas^3^ and has led to novel therapeutic targets being identified^4^. The mutated enzyme produces the metabolite 2-hydroxyglutarate which can be detected both in tissue and *in vivo* providing a non-invasive test for this subgroup illustrating a key advantage for metabolite biomarkers of prognosis.

Whilst the identification of specific prognostic subgroups has advantages, markers that are applicable across multiple tumour types also give clinical value. Histological markers, such as Ki67 proliferation index, are used regardless of diagnosis to assess tumour aggressiveness, and *MYC* status is a marker of poor prognosis used clinically in many different tumour types^5^. Specific metabolites have been proposed as markers of prognosis over a range of different children’s brain tumours using *in vivo* Magnetic Resonance Spectroscopy (MRS). Specifically, glutamine and N-acetylaspartate (NAA) were found to be markers of good prognosis whilst scyllo-inositol and mobile lipids markers of poor prognosis^6^.

However, performing MRS on typical clinical scanners is associated with a number of limitations, in particular, the relatively small number of metabolites that can be measured accurately and the requirement for sampling of relatively large tumour volumes. *Ex-vivo* ^1^H-High-Resolution Magic Angle Spinning (HR-MAS) can analyse small brain tumour tissue samples to provide quantitative information on a larger number of metabolites^7^. HR-MAS has identified prognostic metabolic markers in a number of tumour types including prostate^8^, colorectal^9^, breast^10^, neuroblastoma^11^ and pancreatic adenocarcinomas^12^. A good agreement has been demonstrated between *in vivo* and *ex vivo* methods^13,14^, providing an indication that HR-MAS-visible metabolites accurately reflect the values present *in situ* and are potentially observable *in vivo*. The primary aim of this study was to identify and measure the concentrations of metabolites in a range of paediatric brain tumours using HR-MAS and test the ability of metabolites to predict survival.

## Methods

### Patients

All patients with brain tumours presenting at the Birmingham Children’s Hospital were eligible to be entered into the study. Approval was obtained from the research ethics committee (NRES East Midlands-Derby, 04/MRE04/41) and informed consent was given by parents/guardians. All experiments were performed in accordance with relevant guidelines and regulations. Patients were accrued between January 1998 and May 2016 and followed up until September 2017. A consensus diagnosis was obtained for each patient by a multidisciplinary team of clinicians that included histopathological diagnosis according to Louis *et al*.^15^. Brain tumour tissue, frozen as soon as possible after resection, was requested from the histopathology tissue bank at Birmingham Children’s Hospital. In total, 133 brain tumour samples were released for HR-MAS. Dates of death and clinical information were obtained from the West Midlands Regional Children’s Tumour Registry. Patients were treated with the protocol appropriate for their respective diagnosis. Broadly, this involved maximal safe surgical resection when the tumour was in a favourable location, with adjuvant radiotherapy and/or chemotherapy if necessary.

From the 133 tumour samples available, 19 were excluded from the analysis due to quality reasons or cause of death not being from the tumour diagnosis. The final cohort consisted of 114 primary pre-treatment brain tumour samples including 36 pilocytic astrocytomas (PA), 32 medulloblastomas (MB), 15 ependymomas (EP), 7 glioblastoma multiforme (GBM), 6 atypical teratoid rhabdoid tumours (ATRT), 4 anaplastic astrocytomas (AA), 4 choroid plexus papilloma (CPP), 3 gangliogliomas (GG), 2 atypical choroid plexus papillomas (ACPP), 2 CNS primitive neuroectodermal tumours (PNET), 1 choroid plexus carcinoma (CPC), 1 astroblastoma (AB) and 1 unclassified low grade astrocytic glial tumour. As the tumour tissue was requested before the release of the WHO CNS classification 2016, diagnoses are those specified by the WHO CNS classification 2007^15^. 35 patients died and 79 were alive at the end of the study. The median follow-up time for the whole cohort was 5.31 years (Table 1).

**Table 1:** Clinical information for patients included in this study, organised by tumour diagnosis.

	Pilocytic astrocytoma	Medullo-blastoma	Ependy-moma	Gliobla-stoma	Atypical teratoid rhabdoid tumour	Anaplastic astrocytoma	Choroid plexus papilloma	Ganglio-glioma	CNS primitive neuroectodermal tumour	Atypical choroid plexus papilloma	Choroid plexus carcinoma	Astrobla-stoma	Astrocytic glial tumour	Total
N	36	32	15	7	6	4	4	3	2	2	1	1	1	**114**
Number of events (n)	3	13	3	6	4	3	0	0	1	1	1	0	0	**35**
Gender (male:female)	18:18	26:6	11:4	5:2	4:2	1:3	4:0	1:2	1:1	1:1	1:0	0:1	1:0	**74:40**
Mean age at diagnosis (years)	8.30	7.11	5.53	5.68	1.10	10.49	3.40	11.07	7.24	2.95	5.09	10.18	11.58	**5.93**
Age range (years)	1.2–15.9	1.5–14.6	0.3–16.3	0.03–11.5	0.02–4.6	4.1–15.5	0.4–10.1	8.7–14.9	2.2–12.3	1.6–4.3	N/A	N/A	N/A	**0.03–16.3**
Median survival (years)	4.71	1.82	1.68	0.94	0.31	1.54	N/A	N/A	0.94	1.93	1.39	N/A	N/A	**1.50**
Anatomical location (n)[supratentorial:infratentorial:spinal]	7:29:0	0:32:0	5:9:1	6:1:0	1:5:0	3:1:0	2:2:0	2:1:0	2:0:0	1:1:0	1:0:0	1:0:0	0:1:0	**31:82:1**

### Sample preparation and HRMAS

The frozen samples were stored at −80 °C until used for HR-MAS. Samples were dissected on dry ice and inserted into either a 12 μl or 50 μl HR-MAS rotor with TMSP (Cambridge Biosciences, Cambridge, UK) as a quantification reference and D_2_O (Sigma-Aldrich, Dorset, UK) to fill the allocated rotor volume. Sample weights ranged from 3.4 to 35.8 mg. HR-MAS experiments were performed using a Bruker Avance 500 MHz (11.74 T) NMR spectrometer and a 4 mm 3 channel HR-MAS z-PFG band probe (Bruker, Coventry, UK). The samples were maintained at a temperature of 4 °C, a spin rate of 4800 Hz set for each experiment with 256 or 512 scans acquired depending on the signal-to-noise ratio. A standard 1D NOESY pulse sequence preceded by 2 s of water presaturation was acquired, with a repetition time of 4 s. In addition, a 285 ms Carr-Purcell-Meiboom-Gill (CPMG) pulse sequence was also acquired for each sample to aid in metabolite identification by minimizing broad signal contributions from lipids and macromolecules.

### Processing of HR-MAS data

**Figure 1:**
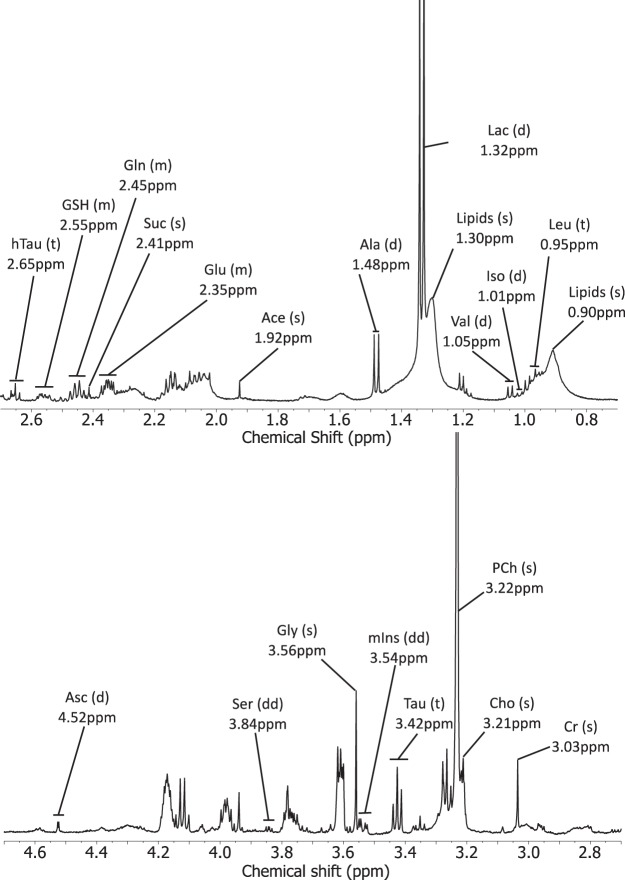
Example spectrum with annotated metabolite resonances and splitting patterns. Not visible in this spectrum are NAA(s) at 2.01ppm, GABA(t) at 2.30ppm, GPC(s) at 3.23ppm, sIns(s) at 3.34ppm and Glc(d) at 4.65ppm. Abbreviations: s, singlet; d, doublet; t, triplet; dd, doublet of doublets; m, multiplet; ppm, parts per million.

Each free induction decay was Fourier transformed using TOPSPIN (Bruker, Coventry, UK). Phase and baseline correction (Whittaker Smoother) were performed using Mnova NMR 9.0 software suite (2014; Mestrelab Research, Spain). Creatine was used as a chemical shift reference at 3.03 ppm. Spectral fitting was then performed between 0.5 and 4.7 ppm. All spectral peaks were deconvoluted and peak integrals measured followed by normalization to TMSP. The following metabolites were quantified and included in the analyses: acetate (Ace), alanine (Ala), ascorbate (Asc), choline (Cho), creatine (Cr), gamma-aminobutyric acid (GABA), glucose (Glc), glutamine (Gln), glutamate (Glu), glutathione (GSH), glycine (Gly), glycerophosphocholine (GPC), hypotaurine (hTau), isoleucine (Iso), lactate (Lac), leucine (Leu), *myo*-inositol (mIns), N-acetylaspartate (NAA), phosphocholine (PCh), serine (Ser), *scyllo*-inositol (sIns), succinate (Suc), taurine (Tau), valine (Val), lipids at 0.9 and lipids at 1.3 ppm (Fig. 1, Supplementary Table 1). Lipids were highly correlated, and so the lipid resonances were summed and termed total lipids. Metabolites were assigned according to Govindaraju *et al*.^16^ whilst lipids were assigned according to Moestue *et al*.^17^. Metabolite concentrations were normalised to the total sum of non-lipid metabolite concentrations.

### Data and statistical analysis

Death caused by the tumour was the endpoint for the study, with time to event calculated from the date of first surgical resection to death. Univariate and multivariate Cox regression were performed on normalised metabolite concentrations and clinical parameters to determine the association of metabolites with survival. Likelihood ratio tests were used to determine significance of Cox models. Clinical parameters found to significantly predict survival in the univariate analyses were included as variables in the multivariate analyses. Univariate P-values were corrected using Benjamini-Hochberg correction to control the false discovery rate.

Validation of the models was performed using leave-one-out cross-validation (LOOCV) of C-indices, as described in Braadland *et al*.^8^. For each individual *i*, a Cox proportional hazards model is created leaving *i* out. The coefficients, β_*i*_, generated are applied to *i*’s covariates, *x*_i_, to obtain a linear predictor *η*_*i*_ = β_*i*_*x*_*i*_. This is repeated for all individuals in the analysis creating the vector *η* = *η*_1_, *η*_2_, *η*_2_*…η*_*n*_, where n is the number of individuals. The resultant vector of linear predictors is used as a single covariate in a Cox proportional hazards model to calculate the C-index. Normalised metabolite concentrations were scaled by dividing each metabolite by its standard deviation prior to univariate and multivariate analysis.

Kaplan-Meier analyses were used to test the difference in survival of patients with brain tumours separated into groups above and below 25%, 50% or 75% quantiles for significant metabolites in the whole cohort. To further test the robustness of the biomarkers, the whole cohort was divided based on the date of tissue collection into an initial cohort (n = 23), comprised of tissue collected during or before June 2006, and a validation cohort (n = 91), comprised of tissue collected after June 2006. The quantile showing the most significant result was recalculated in the initial cohort and the obtained value was applied to the validation cohort. Kaplan-Meier analysis was performed using logrank tests. All survival analyses were performed using the survival library written for the R software package^18^.

## Results

### Univariate cox regression

A total of 25 metabolite features were assigned as quantified using HR-MAS (Fig. 1, Supplementary Table 1). The mean metabolite concentrations for the different tumour groups are given in Supplementary Table 2. Univariate Cox regression identified 2 metabolites with prognostic potential (Table 2, Supplementary Table 3); higher Gln was associated with decreased risk of death, whilst a higher concentration of total lipids was associated with an increased risk of death. Whilst Val, Suc and hTau were significant in the univariate analysis, they were no longer significant upon P-value correction. Of the clinical features tested, diagnosis was shown to be a significant predictor of survival; patients diagnosed with AA, ATRT, CPC, GBM, MB and PNET had a significantly higher risk of death than PA patients (Table 2). Similar to Val, Suc and hTau, age at diagnosis was significant in the univariate analysis but was no longer significant after P-value correction. Gender and tumour location were not significant predictors of survival in this analysis (Supplementary Table 4).

**Table 2:** Univariate and multivariate Cox models for metabolite concentrations demonstrating their ability to predict overall survival of paediatric brain tumour patients.

Variable	Univariate analysis		Multivariate analyses			
	HR (95% CI)	Corrected P-value	Glutamine model		Total lipids model	
			HR (95% CI)	P-value	HR (95% CI)	P-value
Gln	0.52 (0.31, 0.87)	0.041	0.42 (0.21–0.85)	0.015	—	—
Total lipids	2.02 (1.53, 2.66)	2.48 × 10^−4^	—	—	1.91 (1.35, 2.68)	2.15 × 10^−4^
Diagnosis		4.58 × 10^−4^				
PA	Reference	—	Ref	—	Ref	—
AA	13.03 (2.62, 64.65)	0.0017	9.93(2.99, 49.59)	0.005	15.80 (3.16, 79.16)	7.86 × 10^−4^
ACPP	9.27 (0.95, 90.57)	0.056	3.41 (0.32, 36.58)	0.31	8.08 (0.82, 79.10)	0.073
ATRT	17.11 (3.80, 76.95)	2.1 × 10^−4^	8.63 (1.75, 42.61)	0.0081	11.69 (2.38, 57.53)	0.0025
CPC	35.46 (3.52, 357.31)	0.0025	9.48 (0.79, 113.46)	0.076	10.39 (0.96, 112.40)	0.054
EP	2.55 (0.51, 12.63)	0.25	1.34 (0.25, 7.09)	0.73	2.61 (0.53, 12.98)	0.24
GBM	31.72 (7.77, 129.45)	1.5 × 10^−6^	32.73 (7.86, 136.23)	1.64 × 10^−6^	22.29 (5.32, 93.31)	2.14 × 10^−5^
MB	5.91 (1.68, 20.76)	0.0056	2.45 (0.61, 9.89)	0.21	5.71 (1.62, 20.11)	0.0066
PNET	14.12 (1.44, 138.02)	0.023	6.03 (0.58, 63.08)	0.13	21.69 (2.15, 218.62)	0.009

As no deaths were recorded for patients diagnosed with GG, AB, CPP or the unclassified glial tumour, these entries have been excluded from this table.

### Multivariate cox regression

The two metabolites with prognostic potential in the univariate analyses were further evaluated in a multivariate analysis. Each metabolite was assessed with diagnosis as a second covariate. Gln and total lipids were found to be predictors of survival independent of diagnosis, with higher concentrations of Gln predicting better survival, whilst higher concentrations of total lipids were associated with a higher risk of death (Table 2).

### Validation of prognostic markers

Upon validation of the univariate analysis using leave one out cross validation of C-indices, total lipids alone demonstrated a marginally greater predictive accuracy than diagnosis alone. Validation of the multivariate models showed a greater predictive accuracy when either Gln or total lipids were combined with diagnosis when compared with diagnosis alone using the same validation method (Table 3).

**Table 3:** Cross validation of the univariate and multivariate models demonstrate improved predictive accuracy after inclusion of either Gln or total lipid concentrations in addition to diagnosis when compared to diagnosis alone.

Variable	Univariate analysis	Multivariate analysis
	LOOCV C-index	LOOCV C-index
Gln	0.641	0.686
Total lipids	0.670	0.727
Diagnosis	0.666	—

**Figure 2:**
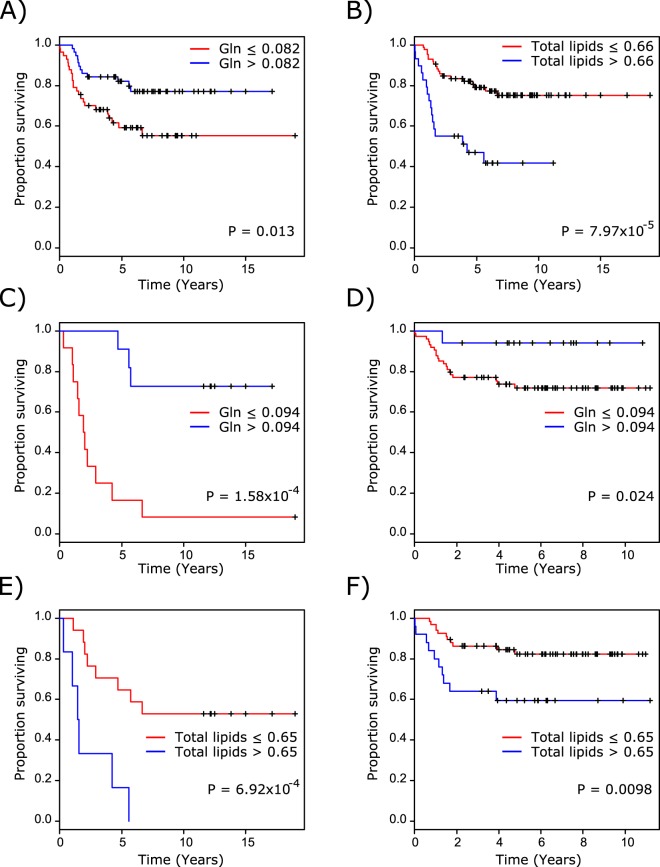
Stratification of patients in the whole cohort into high and low metabolite concentration groups using (**A**) 50% quantile Gln and (**B**) 75% quantile total lipids demonstrates significant differences in survival. Stratification of patients in (**C**) the initial cohort using 50% quantile Gln demonstrates a significant difference in survival which remains upon application of the determined value to (**D**) the validation cohort. Stratification of patients in (**E**) the initial cohort using 75% quantile total lipids demonstrates a significant difference in survival which remains upon application of the determined value to (**F**) the validation cohort.

The relationship between survival and metabolic predictors remained when the whole cohort was stratified into high and low concentrations groups using 25%, 50% and 75% quantiles. The difference in survival between patients with high and low Gln was greatest when cases were stratified by the 50% quantile Gln, whilst the difference between high and low lipids patients was greatest upon stratification using the 75% quantile (Fig. 2A,B). Separation of the whole cohort into an initial cohort and validation cohort further demonstrated the prognostic ability of Gln and total lipids. The difference in survival using the 50% quantile Gln concentration in the initial cohort reached prognostic significance, which was maintained when the cut-off was applied to the validation cohort (Fig. 2C,D). The difference in survival using the 75% quantile total lipids in the initial cohort demonstrated prognostic significance, which remained following application to the validation cohort (Fig. 2E,F).

## Discussion

This study has shown that the concentration of metabolites measured in fresh, frozen diagnostic biopsy tissue from a variety of paediatric brain tumours using HR-MAS varies with disease aggressiveness. Through univariate and multivariate Cox models, it was shown that both Gln and total lipids have prognostic significance independent of tumour diagnosis and that addition of metabolite concentrations can aid in predicting overall survival. Interestingly, through cross-validation, total lipid concentration alone outperformed diagnosis in predicting survival, although the difference was small.

We have previously reported *in vivo* Gln concentration as a predictor of good prognosis in paediatric brain tumours independent of diagnosis^6^, and it is encouraging to have this confirmed by *ex vivo* spectroscopy. Gln is poorly resolved at an MR scanner field strength of 1.5 T due to the overlapping of signals from Glu, NAA and macromolecules. Techniques have been developed to improve the accuracy of Gln quantification; 1D MRS techniques include averaging spectra from different echo times (TE)^19^, the use of optimal TE’s^20-22^, very short TE’s^23,24^ and spectral-selective refocusing^25^. 2D techniques have also been developed but are not favoured due to the high level of expertise required to ensure reliable results and the time taken to acquire the data^26^. There have been recent advancements in ultrafast 2D NMR^27^, although this is still in its infancy. Overall, there is a realistic prospect that it will be possible to routinely measure tumour Gln levels *in vivo* in the near future.

Gln is the most abundant amino acid in blood plasma, and its metabolism has long been known to be important in tumour biology^28^; uptake has been shown to increase in brain tumours in order to support cellular bioenergetics and biosynthetic processes needed for proliferation^29^. Metabolism is under the direct control of major transcription factors such as TP53, Myc and Ras^30^. Mutations or changes in the regulation of these genes are common in cancers, including brain tumours, and switch the cells’ metabolism from oxidative phosphorylation of glucose to energy inefficient glucose fermentation and glutaminolysis^31^. This switch to Gln metabolism has been linked to poor prognosis^32^, therefore Gln concentration may aid stratification of patients into risk groups and inform treatment decisions. Due to the reliance of some cancers on Gln, the metabolism of this amino acid has been identified as a potential target for treatment^33^.

Lipids have also previously been identified as biomarkers of survival through *in vivo* MRS of paediatric brain tumours. The lipid signals can be generated by mobile lipids stored in cytoplasmic lipid droplets^34,35^ or by lipids in areas of tissue necrosis^36,37^, although it is unlikely that highly necrotic areas of tumour would be sampled. The role of lipids droplets in brain tumours is not well understood; however, there is evidence suggesting the number of lipid droplets increases in response to cellular stress and are associated with growth arrest^38^ and necrosis^34^. Furthermore, the number of cells positively stained for adipophilin, a lipid droplet associated protein, was shown to significantly increase with tumour grade^39^.

Risk stratification is not the only potential benefit of enhanced metabolite quantification. Progression of disease and response to treatment can be monitored using MRS. Increases in Gln concentration in contralateral white matter is linked to glioblastoma migration^40^, whilst reductions in the concentration of phosphocholine and lactate in glioblastoma cell lines have been observed after treatment with mTor and PI3K inhibitors^41^, reflecting the observation of reduced total Cho in orthotopic glioblastoma mouse models^42^. Increases in lipid concentrations have been correlated with responses to treatment in cell lines exposed to chemotherapy agents^38,43^.

Suc, hTau and Val, which were significant in the univariate analysis before correction for multiple comparisons, warrant further investigation considering the limited understanding of these metabolites in the context of paediatric brain tumours. Increases in both Suc and hTau were associated with a longer survival time, whilst increases in Val were associated with a shorter survival time.

Despite *in vivo* studies identifying NAA and sIns as prognostic markers, neither metabolite reached prognostic significance in the current study of tumour tissue. In addition to the limited sample size which could explain this, it is worth noting that the population of tumours included is somewhat different to that of *in vivo* studies and this could also contribute to the finding. There is a lack of available frozen tissue from surgically intractable tumours such as optic pathway gliomas and brain stem tumours unlike *in vivo* studies. Optic pathway gliomas have been shown to have a high concentration of NAA and a good prognosis whilst brain stem tumours have a higher concentration of sIns and a poor prognosis^6^.

In conclusion, this study has confirmed that the concentration of Gln and lipids in resected tissue are predictors of survival independent of diagnosis. The importance of Gln and lipids as predictors of prognosis in children’s tumours should lead to their measurement in tumour tissue, the implementation of MRS techniques to accurately measure their concentration non-invasively and the exploration of novel agents which can alter their metabolism for therapeutic effect.

## Supplementary information

Supplementary Information

## Data Availability

The datasets generated and analysed during the current study are available from the corresponding author on reasonable request.
