# Supplementary material for: Ex vivo metabolite profiling of paediatric central nervous system tumours reveals prognostic markers
Source: Sci Rep. 2019 Jul 19;9:10473. doi: 10.1038/s41598-019-45900-x (PMC6642141; doi:10.1038/s41598-019-45900-x)
Supplement: Supplementary file 1 — Supplementary Information [file 41598_2019_45900_MOESM1_ESM.docx]

***Ex vivo* metabolite profiling of paediatric central nervous system tumours reveals prognostic markers**

Christopher D. Bennett, Simrandip K. Gill, Sarah E. Kohe, Martin P. Wilson, Nigel P. Davies, Theodoros N. Arvanitis, Daniel Tennant and Andrew C. Peet

**Supplementary table 1 –** Metabolite assignments including peak location on spectrum and splitting pattern

| **Metabolite** | **^1^H chemical shift (ppm)** | **Multiplicity** |
| --- | --- | --- |
| Lipid | 0.90 | Broad singlet |
| Leucine (Leu) | 0.95 | Triplet |
| Isoleucine (Iso) | 1.01 | Doublet |
| Valine (Val) | 1.05 | Doublet |
| Lipid | 1.30 | Broad singlet |
| Lactate (Lac) | 1.32 | Doublet |
| Alanine (Ala) | 1.48 | Doublet |
| Acetate (Ace) | 1.92 | Singlet |
| N-acetylaspartate (NAA) | 2.01 | Singlet |
| γ-amino butyric acid (GABA) | 2.30 | Triplet |
| Glutamate (Glu) | 2.35 | Multiplet |
| Succinate (Suc) | 2.41 | Singlet |
| Glutamine (Gln) | 2.45 | Multiplet |
| Glutathione (GSH) | 2.55 | Multiplet |
| Hypotaurine (hTau) | 2.65 | Triplet |
| Creatine (Cr) | 3.03 | Singlet |
| Choline (Cho) | 3.21 | Singlet |
| Phosphocholine (PCh) | 3.22 | Singlet |
| Glycerophosphocholine (GPC) | 3.23 | Singlet |
| Scyllo-inositol (sIns) | 3.34 | Singlet |
| Taurine (Tau) | 3.42 | Triplet |
| Myo-inositol (mIns) | 3.54 | Doublet of doublets |
| Glycine (Gly) | 3.56 | Singlet |
| Serine (Ser) | 3.84 | Doublet of doublets |
| Ascorbate (Asc) | 4.52 | Doublet |
| β D-glucose (Glc) | 4.65 | Doublet |

**Supplementary table 2** – mean normalised metabolite concentrations and standard deviations for each tumour type.

| Tumour type | | PA | AA | AB | ACPP | Astro_glial | ATRT | CPC | CPP | EP | GBM | GG | MB | PNET |
| --- | --- | --- | --- | --- | --- | --- | --- | --- | --- | --- | --- | --- | --- | --- |
| N | | 36 | 4 | 1 | 2 | 1 | 6 | 1 | 4 | 15 | 7 | 3 | 32 | 2 |
| Acetate | Mean | 0.0053 | 0.0082 | 0.0004 | 0.0031 | 0.0160 | 0.0042 | 0.0008 | 0.0119 | 0.0016 | 0.0022 | 0.0052 | 0.0036 | 0.0039 |
|  | SD | 0.0076 | 0.0100 | NA | 0.0019 | NA | 0.0021 | NA | 0.0210 | 0.0031 | 0.0025 | 0.0058 | 0.0044 | 0.0055 |
| Ala | Mean | 0.0459 | 0.0148 | 0.0285 | 0.0209 | 0.0459 | 0.0440 | 0.0933 | 0.0408 | 0.0328 | 0.0331 | 0.0451 | 0.0328 | 0.0716 |
|  | SD | 0.0192 | 0.0061 | NA | 0.0128 | NA | 0.0287 | NA | 0.0237 | 0.0184 | 0.0191 | 0.0339 | 0.0240 | 0.0478 |
| Asc | Mean | 0.0145 | 0.0151 | 0.0345 | 0.0109 | 0.0101 | 0.0168 | 0.0216 | 0.0057 | 0.0099 | 0.0190 | 0.0042 | 0.0276 | 0.0189 |
|  | SD | 0.0098 | 0.0040 | NA | 0.0154 | NA | 0.0025 | NA | 0.0049 | 0.0061 | 0.0176 | 0.0073 | 0.0173 | 0.0126 |
| Cho | Mean | 0.0140 | 0.0092 | 0.0102 | 0.0101 | 0.0130 | 0.0133 | 0.0057 | 0.0076 | 0.0108 | 0.0144 | 0.0152 | 0.0131 | 0.0237 |
|  | SD | 0.0084 | 0.0025 | NA | 0.0117 | NA | 0.0053 | NA | 0.0053 | 0.0084 | 0.0084 | 0.0037 | 0.0114 | 0.0157 |
| Cr | Mean | 0.0492 | 0.0655 | 0.0080 | 0.0020 | 0.0196 | 0.0346 | 0.0049 | 0.0087 | 0.0511 | 0.0468 | 0.0792 | 0.0614 | 0.0483 |
|  | SD | 0.0269 | 0.0151 | NA | 0.0029 | NA | 0.0331 | NA | 0.0028 | 0.0242 | 0.0214 | 0.0385 | 0.0268 | 0.0214 |
| GABA | Mean | 0.0033 | 0.0000 | 0.0000 | 0.0105 | 0.0949 | 0.0090 | 0.0000 | 0.0053 | 0.0006 | 0.0011 | 0.0000 | 0.0054 | 0.0238 |
|  | SD | 0.0098 | 0.0000 | NA | 0.0148 | NA | 0.0221 | NA | 0.0105 | 0.0022 | 0.0029 | 0.0000 | 0.0153 | 0.0058 |
| Glc | Mean | 0.0137 | 0.0131 | 0.0000 | 0.0069 | 0.0000 | 0.0034 | 0.0000 | 0.0187 | 0.0068 | 0.0065 | 0.0046 | 0.0109 | 0.0000 |
|  | SD | 0.0194 | 0.0082 | NA | 0.0097 | NA | 0.0082 | NA | 0.0132 | 0.0094 | 0.0156 | 0.0080 | 0.0315 | 0.0000 |
| Gln | Mean | 0.1488 | 0.0924 | 0.0312 | 0.0484 | 0.0543 | 0.0690 | 0.0198 | 0.0430 | 0.0870 | 0.1085 | 0.0855 | 0.0629 | 0.0436 |
|  | SD | 0.0809 | 0.0323 | NA | 0.0102 | NA | 0.0477 | NA | 0.0172 | 0.0430 | 0.0695 | 0.0598 | 0.0279 | 0.0335 |
| Glu | Mean | 0.0557 | 0.0304 | 0.1772 | 0.0748 | 0.0566 | 0.1054 | 0.0273 | 0.0886 | 0.0429 | 0.0585 | 0.1059 | 0.0479 | 0.0836 |
|  | SD | 0.0198 | 0.0201 | NA | 0.0031 | NA | 0.0480 | NA | 0.0129 | 0.0202 | 0.0336 | 0.0672 | 0.0308 | 0.1046 |
| Gly | Mean | 0.0510 | 0.0155 | 0.4026 | 0.0173 | 0.0226 | 0.0430 | 0.0279 | 0.0526 | 0.0428 | 0.0465 | 0.0431 | 0.0687 | 0.0255 |
|  | SD | 0.0321 | 0.0132 | NA | 0.0058 | NA | 0.0156 | NA | 0.0421 | 0.0298 | 0.0232 | 0.0422 | 0.0363 | 0.0116 |
| GPC | Mean | 0.0225 | 0.0185 | 0.0074 | 0.0250 | 0.0284 | 0.0121 | 0.0191 | 0.0248 | 0.0145 | 0.0165 | 0.0463 | 0.0073 | 0.0202 |
|  | SD | 0.0096 | 0.0090 | NA | 0.0353 | NA | 0.0139 | NA | 0.0311 | 0.0082 | 0.0131 | 0.0305 | 0.0089 | 0.0211 |
| GSH | Mean | 0.0059 | 0.0316 | 0.0000 | 0.0270 | 0.0221 | 0.0303 | 0.0220 | 0.0106 | 0.0298 | 0.0293 | 0.0000 | 0.1033 | 0.0205 |
|  | SD | 0.0127 | 0.0122 | NA | 0.0073 | NA | 0.0242 | NA | 0.0072 | 0.0160 | 0.0275 | 0.0000 | 0.0969 | 0.0291 |
| hTau | Mean | 0.0292 | 0.0000 | 0.0000 | 0.0000 | 0.0000 | 0.0065 | 0.0051 | 0.0068 | 0.0111 | 0.0167 | 0.0061 | 0.0153 | 0.0026 |
|  | SD | 0.0237 | 0.0000 | NA | 0.0000 | NA | 0.0074 | NA | 0.0136 | 0.0169 | 0.0169 | 0.0106 | 0.0087 | 0.0037 |
| Iso | Mean | 0.0015 | 0.0000 | 0.0020 | 0.0023 | 0.0000 | 0.0032 | 0.0019 | 0.0017 | 0.0051 | 0.0034 | 0.0000 | 0.0025 | 0.0020 |
|  | SD | 0.0027 | 0.0000 | NA | 0.0032 | NA | 0.0013 | NA | 0.0022 | 0.0160 | 0.0019 | 0.0000 | 0.0031 | 0.0029 |
| Lac | Mean | 0.3384 | 0.2804 | 0.1787 | 0.3550 | 0.4128 | 0.2984 | 0.6122 | 0.4102 | 0.3033 | 0.3478 | 0.3609 | 0.2174 | 0.3770 |
|  | SD | 0.0933 | 0.0758 | NA | 0.1645 | NA | 0.1188 | NA | 0.0857 | 0.0966 | 0.1459 | 0.0475 | 0.0827 | 0.3818 |
| Leu | Mean | 0.0052 | 0.0000 | 0.0087 | 0.0050 | 0.0000 | 0.0080 | 0.0000 | 0.0614 | 0.0278 | 0.0052 | 0.0000 | 0.0070 | 0.0000 |
|  | SD | 0.0082 | 0.0000 | NA | 0.0071 | NA | 0.0077 | NA | 0.1056 | 0.1040 | 0.0112 | 0.0000 | 0.0082 | 0.0000 |
| mIns | Mean | 0.0954 | 0.2527 | 0.0539 | 0.2805 | 0.1006 | 0.0578 | 0.0093 | 0.1075 | 0.1937 | 0.1050 | 0.1247 | 0.0854 | 0.1216 |
|  | SD | 0.0438 | 0.0717 | NA | 0.2198 | NA | 0.0570 | NA | 0.0304 | 0.0959 | 0.0679 | 0.0526 | 0.0566 | 0.0233 |
| NAA | Mean | 0.0185 | 0.0087 | 0.0018 | 0.0033 | 0.0054 | 0.0217 | 0.0000 | 0.0000 | 0.0086 | 0.0045 | 0.0274 | 0.0093 | 0.0000 |
|  | SD | 0.0148 | 0.0084 | NA | 0.0047 | NA | 0.0284 | NA | 0.0000 | 0.0263 | 0.0093 | 0.0219 | 0.0086 | 0.0000 |
| PCh | Mean | 0.0219 | 0.0162 | 0.0171 | 0.0563 | 0.0110 | 0.1016 | 0.0711 | 0.0475 | 0.0315 | 0.0301 | 0.0117 | 0.0836 | 0.0172 |
|  | SD | 0.0172 | 0.0058 | NA | 0.0248 | NA | 0.1022 | NA | 0.0315 | 0.0293 | 0.0182 | 0.0060 | 0.0450 | 0.0016 |
| Ser | Mean | 0.0153 | 0.0747 | 0.0000 | 0.0000 | 0.0000 | 0.0068 | 0.0000 | 0.0000 | 0.0137 | 0.0186 | 0.0000 | 0.0072 | 0.0000 |
|  | SD | 0.0198 | 0.1240 | NA | 0.0000 | NA | 0.0079 | NA | 0.0000 | 0.0128 | 0.0210 | 0.0000 | 0.0092 | 0.0000 |
| sIns | Mean | 0.0043 | 0.0138 | 0.0020 | 0.0067 | 0.0071 | 0.0013 | 0.0019 | 0.0091 | 0.0039 | 0.0013 | 0.0024 | 0.0023 | 0.0049 |
|  | SD | 0.0044 | 0.0142 | NA | 0.0014 | NA | 0.0020 | NA | 0.0019 | 0.0029 | 0.0016 | 0.0022 | 0.0024 | 0.0053 |
| Succinate | Mean | 0.0053 | 0.0019 | 0.0000 | 0.0044 | 0.0003 | 0.0025 | 0.0024 | 0.0013 | 0.0022 | 0.0021 | 0.0017 | 0.0016 | 0.0030 |
|  | SD | 0.0073 | 0.0009 | NA | 0.0043 | NA | 0.0011 | NA | 0.0027 | 0.0013 | 0.0025 | 0.0020 | 0.0014 | 0.0036 |
| Tau | Mean | 0.0275 | 0.0329 | 0.0295 | 0.0235 | 0.0706 | 0.0972 | 0.0492 | 0.0295 | 0.0627 | 0.0712 | 0.0286 | 0.1167 | 0.0747 |
|  | SD | 0.0232 | 0.0307 | NA | 0.0115 | NA | 0.0437 | NA | 0.0244 | 0.0418 | 0.0470 | 0.0339 | 0.0690 | 0.0411 |
| Val | Mean | 0.0076 | 0.0044 | 0.0064 | 0.0062 | 0.0086 | 0.0101 | 0.0046 | 0.0069 | 0.0056 | 0.0116 | 0.0021 | 0.0072 | 0.0134 |
|  | SD | 0.0034 | 0.0055 | NA | 0.0022 | NA | 0.0031 | NA | 0.0023 | 0.0021 | 0.0048 | 0.0037 | 0.0070 | 0.0010 |
| Total lipids | Mean | 0.3638 | 0.2104 | 0.2319 | 0.5957 | 0.1190 | 1.7049 | 1.9007 | 0.7901 | 0.4409 | 1.0145 | 0.1350 | 0.5277 | 0.3448 |
|  | SD | 0.4186 | 0.2394 | NA | 0.4977 | NA | 1.8463 | NA | 0.8331 | 0.3872 | 0.8470 | 0.2338 | 0.3265 | 0.4876 |

**Supplementary table 3 –** Univariate Cox regression results for all metabolites. Abbreviations: BH – Benjamini-Hochberg.

| **Variable** | **HR (95% CI)** | **P-value** | **BH corrected P-values** |
| --- | --- | --- | --- |
| **Ace** | 0.83 (0.52, 1.33) | 0.39 | 0.51 |
| **Ala** | 0.82 (0.57, 1.19) | 0.28 | 0.51 |
| **Asc** | 1.16 (0.86, 1.56) | 0.34 | 0.52 |
| **Cho** | 1.11 (0.83, 1.50) | 0.49 | 0.59 |
| **Cr** | 0.94 (0.67, 1.31) | 0.71 | 0.76 |
| **GABA** | 0.79 (0.48, 1.31) | 0.29 | 0.49 |
| **Glc** | 0.66 (0.36, 1.18) | 0.086 | 0.28 |
| **Gln** | 0.52 (0.31, 0.87) | 0.0042 | 0.041 |
| **Glu** | 1.05 (0.76, 1.45) | 0.78 | 0.81 |
| **Gly** | 0.84 (0.55, 1.29) | 0.38 | 0.52 |
| **GPC** | 0.75 (0.51, 1.09) | 0.11 | 0.32 |
| **GSH** | 1.18 (0.90, 1.54) | 0.25 | 0.48 |
| **hTau** | 0.66 (0.42, 1.03) | 0.04 | 0.17 |
| **Iso** | 1.01 (0.75, 1.35) | 0.96 | 0.96 |
| **Lac** | 0.92 (0.65, 1.30) | 0.64 | 0.74 |
| **Leu** | 0.77 (0.67, 1.62) | 0.34 | 0.49 |
| **mIns** | 1.28 (0.96, 1.70) | 0.12 | 0.32 |
| **NAA** | 0.76 (0.48, 1.18) | 0.17 | 0.41 |
| **PCh** | 1.25 (0.91, 1.72) | 0.19 | 0.42 |
| **Ser** | 1.16 (0.90, 1.49) | 0.33 | 0.53 |
| **sIns** | 0.92 (0.63, 1.36) | 0.68 | 0.76 |
| **Suc** | 0.45 (0.19, 1.03) | 0.018 | 0.10 |
| **Tau** | 1.31 (1.02, 1.68) | 0.053 | 0.19 |
| **Val** | 1.55 (1.17, 2.06) | 0.0077 | 0.06 |
| **Total lipids** | 2.02 (1.53, 2.66) | 1.71x10^-5^ | 0.00025 |

**Supplementary table 4** – Univariate Cox regression results for clinical factors. Abbreviations: BH – Benjamini-Hochberg.

| **Variable** | **HR (95% CI)** | **P-value** | **BH corrected P-values** |
| --- | --- | --- | --- |
| **Gender** | 1.62 (0.76, 3.45) | 0.20 | 0.40 |
| **Age** | 0.92 (0.85, 0.99) | 0.023 | 0.099 |
| **Diagnosis** | - | 1.58x10^-5^ | 4.58x10^-4^ |
| **Location** | - | 0.40 | 0.52 |
